# Supplementary material for: An open-source framework for large-scale, flexible evaluation of biomedical text mining systems
Source: J Biomed Discov Collab. 2008 Jan 29;3:1. doi: 10.1186/1747-5333-3-1 (PMC2276192; doi:10.1186/1747-5333-3-1)
Supplement: Additional file 1 — Evaluation platform source code. The additional file contains the software evaluation framework discussed in this paper. The most current release can be downloaded from SourceForge.net [1]. See the accompanying README file for instructions on installation and use. Also included in the distribution is a collection of UIMA wrappers for some commonly used BioNLP tools and annotated corpora. [file 1747-5333-3-1-S1.zip › bionlp_uima_repository-1.0.1/docs/api/edu/uchsc/ccp/util/nlp/mention/comparison/MentionComparator.html]

MentionComparator (Center for Computational Pharmacology Util/UIMA API)


|  |  |  |
| --- | --- | --- |
| **Overview** \| **Package** \| **Class** \| **Tree** \| **Deprecated** \| **Index** \| **Help** | |  |
| **PREV CLASS**   **NEXT CLASS** | **FRAMES**    **NO FRAMES**     **All Classes** |  |
| SUMMARY: NESTED \| FIELD \| CONSTR \| METHOD | DETAIL: FIELD \| CONSTR \| METHOD |  |


---


## edu.uchsc.ccp.util.nlp.mention.comparison Class MentionComparator

```
java.lang.Object
  edu.uchsc.ccp.util.nlp.mention.comparison.MentionComparator
```

**All Implemented Interfaces:**: Comparator<Mention>

**Direct Known Subclasses:**: IdenticalMentionComparator

---

``` public abstract class MentionComparator extends Object implements Comparator<Mention> ```

This is an abstract class for mention comparators. Each level of comparison stops at the class mention level.  
Level 0: comparison of the classmention names.  
Level 1: comparison of the classmention names, and optionally their text annotation spans, and if they match, a comparison of the slotmentions and
their slot values, and a comparison of complexslotmentions and their classmentionnames  
Level 2: Comparison of the class mentions in level in the previous level that were only compared by mention name  
...  
Level N: Comparison of the class mentions in level in the previous level that were only compared by mention name  
  
Example:  
TA1  
--- CM1  
------ SM1a  
---------SV1a  
------ SM1b  
---------SV1b  
------ CSM1a  
--------- CM2 ==> TA2  
------------ SM2a  
--------------- SV2a  
------------ CSM2a  
--------------- CM3 ==> TA3  
------ CSM1b  
--------- CM4 ==> TA4  
------------ CSM4a  
-------------- CM5 ==> TA5  
  
Level 0: TA1 span and CM1 mention name compared  
Level 1: SM1a name and values, SM1b name and values, CM2 name, TA2 span, CM4 name, and TA4 span are also compared  
Level 2: SM2a name and values, CM3 name, TA3 span, CM5 name, TA5 span  
Level 3: CM3 and CM5 slots if there are any   

**Author:**
:   williamb

---

| **Field Summary** | |
| --- | --- |
| `protected  int` | `MULTIPLIER`             So that differences found in mentions can be distinguished between differences found in spans, the mention comparators should return -3, 0, or 3, therefore the MULTIPLIER variable is used to scale the compare() output. |


**Constructor Summary**

`MentionComparator()`


| **Method Summary** | |
| --- | --- |
| `int` | `compare(Mention mention1, Mention mention2)`             The default use of compare() will compare all depth levels of the mention heirarchy, and will compare any linked TextAnnotations using the StrictSpanComparator. |
| `abstract  int` | `compare(Mention mention1, Mention mention2, SpanComparator spanComparator, int maximumComparisonDepth, int depth)` |
| `protected  boolean` | `equalComplexSlotMentions(List<ComplexSlotMention> csmList1, List<ComplexSlotMention> csmList2, SpanComparator spanComparator, ComplexSlotMentionComparator csmComparator, int maximumComparisonDepth, int depth)`             This method compares two lists of complex slot mentions by iterating through them. |
| `protected  boolean` | `equalSlotMentions(List<SlotMention> smList1, List<SlotMention> smList2, SpanComparator spanComparator, SlotMentionComparator slotMentionComparator, int maximumComparisonDepth, int depth)`             This method compares two lists of slot mentions by iterating through them. |
| `abstract  boolean` | `hasEquivalentMentionNames(Mention mention1, Mention mention2)` |

**Methods inherited from class java.lang.Object**

`clone, equals, finalize, getClass, hashCode, notify, notifyAll, toString, wait, wait, wait`

**Methods inherited from interface java.util.Comparator**

`equals`

**Field Detail**

### MULTIPLIER

```
protected final int MULTIPLIER
```

:   So that differences found in mentions can be distinguished between differences found in spans, the mention comparators should return -3, 0, or
    3, therefore the MULTIPLIER variable is used to scale the compare() output.

    **See Also:**: Constant Field Values


**Constructor Detail**

### MentionComparator

```
public MentionComparator()
```


**Method Detail**

### compare

```
public int compare(Mention mention1,
                   Mention mention2)
```

:   The default use of compare() will compare all depth levels of the mention heirarchy, and will compare any linked TextAnnotations using the
    StrictSpanComparator. This is specified by a maximum depth of -1.  
      
    So that differences found in mentions can be distinguished between differences found in spans, the mention comparators should return -3, 0, or
    3 (instead of -1, 0, or 1)

    :   **Specified by:**: `compare` in interface `Comparator<Mention>`
    :   **Parameters:**: `mention1` -: `mention2` -

---


### compare

```
public abstract int compare(Mention mention1,
                            Mention mention2,
                            SpanComparator spanComparator,
                            int maximumComparisonDepth,
                            int depth)
```

---


### hasEquivalentMentionNames

```
public abstract boolean hasEquivalentMentionNames(Mention mention1,
                                                  Mention mention2)
```

---


### equalSlotMentions

```
protected boolean equalSlotMentions(List<SlotMention> smList1,
                                    List<SlotMention> smList2,
                                    SpanComparator spanComparator,
                                    SlotMentionComparator slotMentionComparator,
                                    int maximumComparisonDepth,
                                    int depth)
```

:   This method compares two lists of slot mentions by iterating through them. A quick check of the number of slot mentions in each list is made at
    first to see if the individual comparisons can be avoided.

---


### equalComplexSlotMentions

```
protected boolean equalComplexSlotMentions(List<ComplexSlotMention> csmList1,
                                           List<ComplexSlotMention> csmList2,
                                           SpanComparator spanComparator,
                                           ComplexSlotMentionComparator csmComparator,
                                           int maximumComparisonDepth,
                                           int depth)
```

:   This method compares two lists of complex slot mentions by iterating through them.


---


|  |  |  |
| --- | --- | --- |
| **Overview** \| **Package** \| **Class** \| **Tree** \| **Deprecated** \| **Index** \| **Help** | |  |
| **PREV CLASS**   **NEXT CLASS** | **FRAMES**    **NO FRAMES**     **All Classes** |  |
| SUMMARY: NESTED \| FIELD \| CONSTR \| METHOD | DETAIL: FIELD \| CONSTR \| METHOD |  |


---
